# The Perception of Evidence for Venous Thromboembolism Prophylaxis Current Practices after Cardiac Surgery: A Canadian Cross-Sectional Survey

**DOI:** 10.1155/2015/795645

**Published:** 2015-11-02

**Authors:** Hani N. Mufti, Roger J. F. Baskett, Rakesh C. Arora, Jean-Francois Légaré

**Affiliations:** ^1^Division of Cardiac Surgery, Department of Surgery, Dalhousie University, Queen Elizabeth II Health Sciences Center, 1796 Summer Street, Room 2269, Halifax, NS, Canada B3H 3A7; ^2^St. Boniface Hospital, I.H. Asper Clinical Research Institute, University of Manitoba, CR3012-369 Tache Avenue, Winnipeg, MB, Canada R2H 2A6

## Abstract

*Background.* Venous thromboembolism (VTE) is the third leading cause of cardiovascular death in patients undergoing surgery. However, VTE prophylaxis practices in cardiac surgery are based on noncardiac surgical literature. The objective of our study was to extract current patterns of VTE prophylaxis practices in cardiac surgery patients. We also aimed to identify health care professionals knowledge of available evidence supporting VTE prophylaxis in adult cardiac surgery patients.* Methods.* A web-based survey was developed and sent to all Canadian cardiac surgery centers with the intent to have the survey distributed to all personnel involved in the perioperative care of adult cardiac surgery patients. Participation in the questionnaire was voluntary and anonymized.* Results.* Thirty-five responses were obtained. Sixty-nine percent reported having an established protocol for VTE prophylaxis. However, 83% reported using VTE prophylaxis in their daily practice despite lack of protocol. The majority (60%) believed that the class of recommendation was high despite the lack of evidence.* Conclusions.* Our survey demonstrated the following. (a) Majority of Canadian centers employ VTE prophylaxis, with considerable variability. (b) There is a misconception among health care professionals about the strength of evidence supporting VTE prophylaxis in cardiac surgery. Our findings highlight the need for appropriately designed studies to fill this knowledge gap.

## 1. Introduction

Venous thromboembolism (VTE), encompassing both deep vein thrombosis (DVT) and pulmonary embolism (PE), is the third leading cause of cardiovascular death, following myocardial infarction (MI) and stroke. Autopsy studies show that VTE remains a leading cause of preventable in-hospital mortality, and that it is often undiagnosed antemortem [1]. This has led to the widespread implementation of VTE prophylaxis guidelines for most patients that are hospitalized for noncardiac surgical interventions [2].

The incidence and the significance of VTE in cardiac surgery patients have not been well defined; neither has VTE prevention after cardiac surgery been well studied. The diagnosis of perioperative VTE in patients undergoing cardiac surgery is often overlooked due to the lack of prospective studies describing this complication, as well as the perception of a low incidence of VTE in these patients [3]. Most cardiac surgery patients have multiple VTE risk factors and may have delayed mobilization after surgery. Up to 13% of patients who undergo coronary arteries bypass surgery or valve replacement will develop radiological evidence of DVT despite following the 8th edition of the ACCP guidelines for optimal thromboprophylaxis and supplementing pharmacological thromboprophylaxis with physical thromboprophylaxis (e.g., bilateral lower extremity intermittent pneumatic compression) devices and early postoperative mobilization [4].

Current VTE prevention practices are largely based on extrapolations from critical care research [2, 5–7] and other surgical literature works (primarily high-risk general surgery and orthopedics) [1, 7–10]. Current recommendations state that, for patients undergoing coronary artery bypass graft surgery, these patients are considered to be high-risk for VTE and prophylaxis should be initiated postoperatively, at least mechanically with or without a pharmacological intervention added, until they are independently mobile [2]. Other cardiac procedures, such as valve replacement or repairs, were not included in the guidelines because the authors assume that they generally require postoperative therapeutic anticoagulation, and VTE rates have not been prospectively assessed in these patients [2].

The primary objective of this survey was to identify the current VTE prophylaxis practices in adult patients undergoing cardiac surgery and determine practitioner's knowledge of currently available evidence that is specific to cardiac surgery patients across Canadian centers.

## 2. Material and Methods

### 2.1. Questionnaire Development

A questionnaire was generated based on a review of the currently available literature and discussion among the investigators. The questions were based in part on a prior survey instrument for intensivists caring for medical-surgical intensive care unit (ICU) patients [5]. The original questionnaire underwent face (logical and information flow), content, and construct validity. Modifications were made to adapt the questionnaire to the cardiac surgery population and to improve item clarity to suit our objective. The domains of interest were respondent demographics, institutional approaches, stated practices, perceived burden of illness of VTE, and respondent's knowledge pertaining to VTE prophylaxis.

### 2.2. Questionnaire Formatting

In the introductory cover letter, we provided the rationale for this questionnaire, specified our primary objective, and previewed the forthcoming survey. The survey consisted of 18 questions. VTE was defined as DVT or PE that occurs in the perioperative period and up to 3 months after surgery. We started the survey by asking the respondents if they are using VTE prophylaxis (yes/no) and, if so, whether they are aware of a protocol at their institute (yes/no).

After that, we tried to probe and understand the respondents' knowledge about VTE and its prophylaxis in adult cardiac surgery patients. We asked respondents to estimate the incidence of DVT and PE in their patients as percentages (0–0.9, 1–5, 6–10, 11–15, 16–20, or >20). We were also interested in what the respondents believed the level of evidence (A, B, C, or unknown) and class of recommendation (I, IIa, IIb, III, IV, or unknown) was for VTE prophylaxis specifically for adult cardiac surgery patients.

We then attempted to extract daily practice patterns across different institutions. We asked the respondents which pharmacological method of VTE prophylaxis they are using (heparin, dalteparin, enoxaparin, fondaparinux, or none) and allowed them to add other drugs or methods (like pneumatic compression) that they use. Since the initiation and duration of prophylaxis has been shown previously to be of significant importance [11, 12], we asked the respondents when they began (pre-op, day of surgery, day 1 post-op, and day 2 post-op, as well as whether the patient was still in the ICU or discharged) and discontinued prophylaxis (mobilizing with one person, mobilizing independently, when central line was removed, or on the day of discharge).

At the end of the survey, we focused on trying to understand who our respondents were and the characteristics of their work environment. We asked the respondents for some basic demographics (age, according to decade to ensure anonymity, and sex), information on where they manage patients (ICU, intermediate medical care unit (IMCU), and/or regular ward), their basic specialty (cardiac surgery, general surgery, anesthesia, internal medicine, family medicine, or physician assistant/nurse practitioner) and subspecialty (critical care, cardiology, cardiac anesthesia, cardiac surgery subspecialty, resident in training, or others), the setting of their ICU (cardiac surgery ICU, mixed cardiac ICU, surgical ICU, mixed general ICU, or others), the ICU model (open, semiopen, or closed), their current title (staff cardiac surgeon, staff intensivist, trainee, or others), their institutional affiliation (academic or community), and their province.

### 2.3. Instrument Administration

In Canada, there are 31 centers where wide ranges of cardiac surgical procedures are offered. Different health care centers can range in capabilities from straightforward uncomplicated coronary artery bypass surgeries to complex aortic reconstructions and heart transplants. Some centers will have a dedicated cardiac ICU and others might not. The ICU coverage model is also heterogeneous. Due to this variability and to achieve a representative sample, we were aiming to have at least a single response from each center, a total of 31 centers across Canada.

We sent a web-based electronic survey (http://www.surveymonkey.com/; SurveyMonkey.com, LLC; Palo Alto, California, USA) entitled “Trends in Venous Thromboembolism (VTE) Prophylaxis in Adult Patients Undergoing Cardiac Surgery Across Canada” to all Canadian cardiac surgery center directors and cardiac intensive care unit directors (see Appendix A in Supplementary Material for a sample of the survey questions, available online at http://dx.doi.org/10.1155/2015/795645). We requested that the survey be distributed to all personnel involved in the perioperative care of adult cardiac surgery patients (including surgeons, intensivists, trainees, and nurse practitioners). The survey remained open for 8 weeks (April 20–June 20, 2012), and we sent email reminders every Wednesday morning, Eastern Standard Time, containing a link to the survey. Participation was voluntary. Agreeing to participate in the survey implied consent, as the cover letter clearly indicated that the results would be published. Individual responses were kept confidential, the data did not include any personal or identifiable information, and the questions were constructed to insure anonymity. The primary focus of this research was the participant's opinions, knowledge, and awareness of the available evidence.

### 2.4. Statistical Analysis

Statistical analysis was completed using Statistical Analysis System version 9.3 (SAS Institute, Cary, NC). Data was reported largely in the form of percentage of respondents. The results of this survey are mainly descriptive of the current patterns of practices across Canada and the health care providers perceptions and knowledge on VTE prophylaxis evidence. Statistical analysis was performed using the Fisher exact test to evaluate the significance of some of the survey results. Values of *P* < 0.05 were considered significant.

## 3. Results

**Figure 1:**
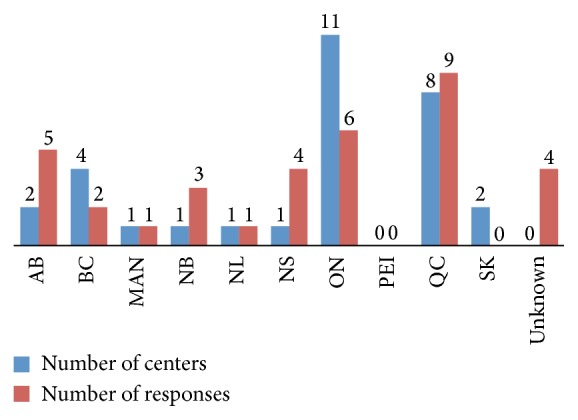
Number of Canadian cardiac surgery centers and respondents by province (AB: Alberta, BC: British Colombia, MB: Manitoba, NB: New Brunswick, NS: Nova Scotia, NL: Newfoundland and Labrador, PEI: Prince Edward Island, ON: Ontario, QC: Quebec, and SK: Saskatchewan).

Over the 8 weeks duration of the survey, we received 35 responses. We did not ask the respondents to specify the center they are working in, to ensure anonymity. In some provinces, the number of responses was less than the number of centers (e.g., Ontario) while in other provinces the number of responses was more than the number of centers (e.g., Alberta) (Figure 1). Because all Canadian cardiac surgery center directors and cardiac intensive care unit directors were asked to distribute the survey, we estimate that the survey reached at least 60 to 70 participants. Unfortunately, we do not have the data to support that.

The respondents were mostly male (91%), primarily between 31 and 50 years of age (60%), employed in an academic/university-affiliated hospital (90%), mostly attending physicians (84%), and primarily managing patients in the ICU (78%).

**Figure 2:**
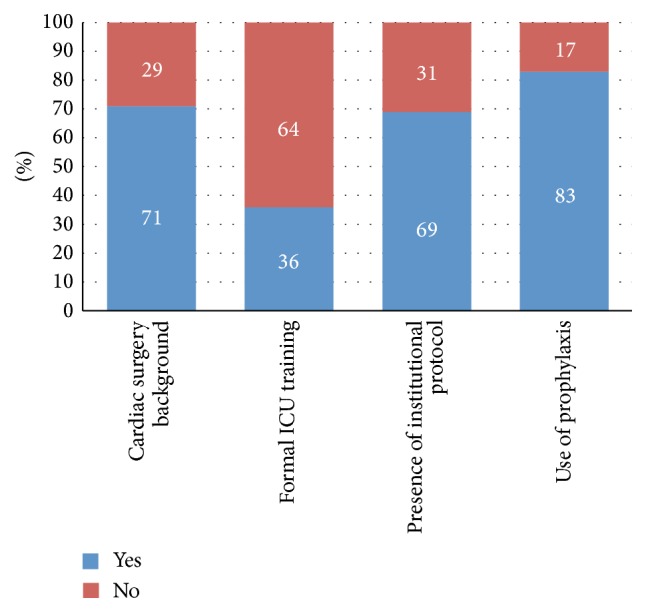
Information about the respondents specialty background, ICU training, presence of institutional protocol, and use of prophylaxis.

Of the centers that responded, 83% of the overall respondents are currently using VTE prophylaxis in their daily practice. However, only 69% reported having an established protocol for the use of VTE prophylaxis in their adult cardiac surgery patients. Most of the respondents were from a cardiac surgery background (71%) but only 36% of the participants underwent a formal ICU training (Figure 2).

Almost 48% of respondents will use unfractionated heparin as their primary method of pharmacological VTE prophylaxis after cardiac surgery. The second most commonly used pharmacological prophylaxis was low molecular weight heparin (dalteparin or enoxaparin ~33%) and only 7.5% used fondaparinux as their primary method of pharmacological prophylaxis. 10% of respondents used other drugs, doses, or methods (tinzaparin 3500 units daily, heparin 5000 units three times a day, and thromboembolic deterrence/intermittent pneumatic mechanical compression stoking) and 2.5% did not start VTE prophylaxis.

**Figure 3:**
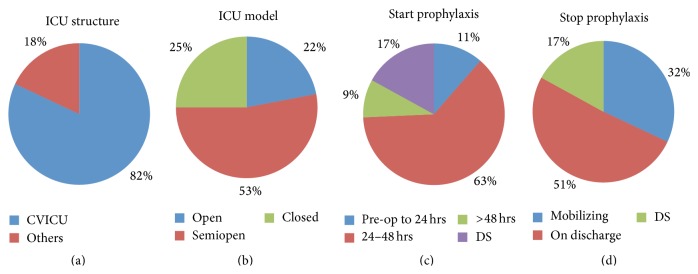
Information about the intensive care unit (ICU) and timing of VTE prophylaxis. (a)   What is the ICU structure? (CVICU: cardiovascular ICU; others: including coronary care unit (CCU) and medical/surgical ICU). (b) What is the ICU model? (c) When do participants start VTE prophylaxis? (pre-op to 24 hrs: preoperative to within the first 24 hours from surgery, 24–48 hrs: 24 to 48 hours from surgery, >48 hrs: more than 48 hours after surgery, and DS: does not start VTE prophylaxis). (d) When do participants stop VTE prophylaxis? (mobilizing: when the patient is mobilizing with no assistance; DS: does not start VTE prophylaxis).

The most common ICU model was semiopen (43%) and 81% of respondents had a dedicated cardiovascular intensive care unit (CVICU) in their institution. Almost two-thirds of the respondents (63%) will start VTE prophylaxis within 24–48 hours after surgery and half of them will discontinue it when the patient gets discharged (51%) (Figure 3).

**Figure 4:**
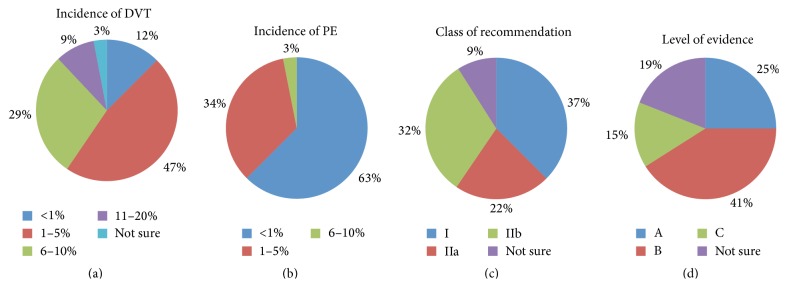
Assesment of participants knowledge and perception of the prevelance VTE after cardiac surgery and the evidance of its prophlaxis. (a) Perception of the incidence of DVT after cardiac surgery. (b) Perception of the incidence of PE after cardiac surgery. (c) Perception of the class of the recommendation of VTE prophylaxis after cardiac surgery (class I: benefit greatly exceeds the risk and treatment should be administered (is effective), class IIa: benefit exceeds the risk and it is reasonable to administer treatment (most likely effective), and class IIb: benefit probably exceeds the risk and treatment may be considered (efficacy less well established)). (d) Perception of the level of evidence of VTE prophylaxis after cardiac surgery (level A: evidence from multiple randomized trials or meta-analysis, level B: limited evidence from a single randomized trial or nonrandomized studies with some conflicting evidence of benefit, and level C: expert opinions or case reports).

With regard to respondents' knowledge on the quality of evidence of VTE prophylaxis in cardiac surgery, 60% believed that the class of recommendation was high (class I and class IIa) but thought that the level of evidence was low (level B or level C). The majority of the respondents believed that the incidence of postoperative above knee DVT in cardiac surgery patients is between 1 and 5% (47%) and the incidence of postoperative pulmonary embolism is less than 1% (62.5%) (Figure 4).

On further examination (see Table 1), we noticed that having a standardized protocol of VTE prophylaxis has a significant effect for starting VTE prophylaxis (*P* ≤ 0.05) (odds ratio = 2.2, 95% Confidence Interval = 1.5–4.2). Subspecialization in surgical versus medical training (cardiology, ICU, and anesthesia) exhibited a positive trend, with 100% of the surgical subspecialty respondents initiating VTE prophylaxis compared to only 72% of the medical subspecialty respondents, but did not reach a statistical significance on initiating prophylaxis (*P* = 0.058) (odds ratio = 1.4, 95% Confidence Interval = 1–1.9). Both groups agreed that the incidence of PE is low (<5%). Gender had no influence on the choice of administration of VTE prophylaxis, although it is important to keep in mind that >90% of our respondents were male. Age, basic specialty, and being in an academic institute had no influence.

## 4. Discussion

Without VTE prophylaxis, objectively confirmed hospital-acquired DVT is approximately 10–40% among medical and surgical patients [13]. In many of these patients, VTE is the most common serious morbidity and one of the factors that are associated with prolonged hospital stay. Not surprisingly, 10% of all preventable in-hospital mortalities are believed to be attribute to fatal PE [13].

The benefit of heparin prophylaxis for the prevention of VTE in noncardiac surgery patients has been illustrated by many studies, with reported 50–70% reduction in DVT incidence [2]. The incidence of radiological evidence of proximal DVT after cardiac surgery can be up to 15%, but less than 2% was clinically detectable [3]. A large retrospective study of 92,699 patients using an administrative database found that the incidence of VTE up to 6 weeks after CABG was similar between the patients who did and did not receive prophylaxis (chemical, mechanical, or both) with an overall incidence of VTE of 0.74% in the entire cohort [14]. However, the authors observed no significant increase in bleeding risk with the use of any of VTE prophylaxis methods [14]. Based on our survey, many of the health professionals caring for cardiac surgery patients perceive that there is a low incidence (between 1 and 5%) of DVT (47% of respondents) but only 12% of the health professionals believe that it is less than 1%. Contrary to this perception, however, the estimated incidence of DVT may be up to 20% and 4% for PE [3].

In a best evidence article by Close et al. [3] a systemic review of the literature was conducted and 5 important points were emphasized: (a) cardiac surgery patients should be considered a high-risk population, (b) the incidence of postoperative VTE is similar to high-risk general surgery patients, (c) this patient population lacks solid evidence, (d) postoperative prophylaxis does not increase the incidence of postoperative bleeding, and (e) postcardiac surgery patients should receive prophylaxis, provided that there are no contraindications.

VTE is an important health care concern that can lead to significant morbidity, mortality, and resource expenditure. According to the “Seventh ACCP Conference on Antithrombotic and Thrombolytic Therapy: Prevention of Venous Thromboembolism Guidelines,” surgical patients who are at highest risk for VTE have a 10–20% chance of having DVT without proper prophylaxis [13]. Based on VTE risk in surgical patients classification, patients undergoing cardiac surgical procedures are considered to be at least high-risk. The “Prevention of Venous Thromboembolism American College of Chest Physicians Evidence-Based Clinical Practice Guidelines (8th Edition)” has a dedicated section on coronary artery bypass surgery (CABG), clearly stating that the need for prophylaxis remains controversial [2]. The guideline clearly acknowledges the lack of well-designed studies and an overall poor body of evidence for this patient population [2]. Unfortunately, our survey illustrates that 59% of the health care professionals believed that the recommendation class is high, with 37% claiming its efficacy (class I) and 22% reasoning that it is most likely effective (class IIa). Interestingly, 41% recognized that there is some conflicting evidence about the efficacy of VTE prophylaxis in this patient population (level of evidence is moderate = level B).

Regrettably, the guidelines only addressed CABG in their recommendation and excluded all other cardiac procedures (e.g., valve replacement and aneurysm surgery) because “they generally require postoperative therapeutic anticoagulation, and VTE rates have not been prospectively assessed in these patients” [2]. We argue that this statement may not be correct, as more bioprosthetic (biological) valves are being implanted than mechanical valves, especially in the aging elderly population. Going back to the guidelines, the recommendations for CABG are based on grade 1C, which is a strong recommendation though it is based on low-quality or very low-quality evidence (observational studies or case series) [15].

Current practices in the United States and Europe have been shifting to more liberal use of bioprosthetic rather than mechanical valves, especially in younger patients [16]. According to a recent publication by Dunning et al., there was a large increase in the annual volume of aortic valve replacement in 2011, and almost 78% of these patients received a bioprosthetic valve [17]. The risk of pericardial effusion and postoperative bleeding is around 4%; however, there is no evidence that starting heparin for VTE prophylaxis the first day after surgery will increase the risk for postoperative bleeding [3]. In a large retrospective study by Kulik et al., the incidence of postoperative bleeding was similar among patients who did or did not receive prophylaxis [14]. While the cardiac surgery community recognizes the importance of VTE prophylaxis, there was no recent attempt to investigate current practice patterns and evaluate health care provided knowledge about the existing evidence.

An interesting finding of our survey was that the presence of an institutional protocol might have considerably prompted the respondent's choice of starting VTE prophylaxis after cardiac surgery. While this might be interesting, it does not indicate causality and it might just be a coincidence. When we wanted to determine the presence of a common standard of daily practices across Canada, we noticed that there is still some variability in prophylaxis practices and the lack of a common standard. Also, we tried to examine the knowledge of the participants about the available evidence; there was a considerable misunderstanding of the strength and level of the evidence. Although there was a trend towards more liberal use of VTE prophylaxis in respondents with surgical training, it did not reach statistical significance. Our study highlights how variable is the management of a preventable yet potentially devastating complication, such as VTE in cardiac surgery patients, due to the lack of robust research that focuses on this high-risk population.

The aim of any survey is to gather reliable and unbiased data from a representative sample of respondents [18]. The study has several limitations, the most notable being the small sample size of personnel dedicated to caring for cardiac surgery patients. Other limitations include the following. (a) The external validity of the survey is questionable, since it was applied on a small sample in a publicly funded health care system. (b) The main objective of survey was testing the knowledge and awareness of the respondents; this might be influenced by personal practices, opinions, and other factors that we failed or were not able to capture with the survey. (c) To ensure anonymity, we did not ask participants for their personal information (like name and city of practice). By doing so, we were not able to determine if we had double responses. (d) Because we were not able to directly contact participants, we cannot be certain how many potential participants did our survey reach, although we estimate that our survey has reached at least 60 potential participants. (e) In our survey, we did not specify the type of procedure being performed. The choice and timing of VTE prophylaxis might have been greatly influenced by the procedure performed and the patient risk of bleeding.

## 5. Conclusions

VTE is a devastating and debilitating complication that has a major impact on patients' health and leads to an added burden on the health care system, especially in terms of overall spending. Prevention via adherence to well-designed prophylaxis protocols is very important, and prophylaxis after surgery is an integral part of the complex process of care for surgical patients. Well-structured and documented evidence of establishing prophylaxis protocols exists for most major specialties (orthopedic, gynecology, trauma, and critical care) but not for cardiac surgery.

Our survey has highlighted the following: (a) While the majority of Canadian centers employ VTE prophylaxis in adult cardiac surgery patients, the used methods and timing are considerably variable. (b) There is a misconception among health care professionals about the strength of evidence supporting VTE prophylaxis in cardiac surgery. (c) The presence of an institutional protocol might promote and support VTE prophylaxis practices.

Our findings highlight the need for appropriately designed studies to fill this knowledge gap with regard to the quality of evidence, the appropriate method for VTE prophylaxis, and its impact on quality of care (safety, complications, and cost).

## Key Messages

1. Majority of Canadian centers employ VTE prophylaxis in adult cardiac surgery patients. The used methods and timing are considerably variable, which are mainly based on personal preferences and the false perception about the availability of solid evidence.
2. There is lack of solid evidence of VTE prophylaxis in adult cardiac surgery patients and the misperception of the quality of available evidence in this high-risk population.
3. The presence of institutional protocols and pathways might help in standardizing and promoting VTE prophylaxis practices after cardiac surgery.

## Supplementary Material

The supplementary material contains a sample of the Survey questions with all possible answers to the questions.

## Figures and Tables

**Table 1:** Relationship between VTE prophylaxis use and different responses.

Response	Used VTE prophylaxis (%)		*P* value
	Yes	No	
Incidence of DVT after cardiac surgery			
<5%	55	6	0.35
>5%	29	10	
Incidence of PE after cardiac surgery			
<5%	82	16	1
>5%	4	0	
Class of recommendation			
Class I or class IIa	53	7	0.37
Class IIb	31	9	
Level of evidence			
Level A or level B	25	0	0.29
Level C	60	15	
Presence of VTE protocol			
Yes	69	0	*<0.05* ^*∗*^
No	14	17	
Influence of basic specialty			
Surgical	61	10	0.61
Medical	22	7	
Influence of subspecialization			
Surgical	42	0	*0.058* ^+^
Medical	42	16	
Gender			
Male	78	6	0.41
Female	42	4	

^*∗*^Statistically significant.

^+^Not statistically significant but a positive trend towards significance.

Basic specialty: surgical = cardiac surgery and general surgery; medical = anesthesia, internal medicine, family medicine, and others.

Subspecialty: surgical = cardiac surgery subspecialty; medical = intensive care, cardiology, cardiac anesthesia, and others.

## Abbreviations

CVICU: Cardiovascular intensive care unit
DVT: Deep vein thrombosis
ICU: Intensive care unit
IMCU: Intermediate care unit
PE: Pulmonary embolism
VTE: Venous thromboembolism
CABG: Coronary arteries bypass surgery.
