# Supplementary material for: The Perception of Evidence for Venous Thromboembolism Prophylaxis Current Practices after Cardiac Surgery: A Canadian Cross-Sectional Survey
Source: Thrombosis. 2015 Nov 2;2015:795645. doi: 10.1155/2015/795645 (PMC4644839; doi:10.1155/2015/795645)
Supplement: Supplementary file 1 — The supplementary material contains a sample of the Survey questions with all possible answers to the questions. [file 795645.f1.doc]

VTE Trends Appendix A
